# Evaluating research-grade and commercial SFDI platforms for burn severity assessment and feature reduction

**DOI:** 10.64898/2026.07.27.741081

**Published:** 2026-07-28

**Authors:** Christopher A. Campbell, Gordon T. Kennedy, Alberto Martín-Pérez, Theresa L. Chin, Victor Joe, Robert J. Christy, Anthony J. Durkin

## Abstract

Spatial frequency domain imaging (SFDI) has demonstrated the ability to provide early, quantitative assessment of burn wound severity. Previous studies using a research-grade SFDI platform showed that high-dimensional datasets incorporating multiple spatial frequencies and wavelengths can predict healing outcomes in controlled porcine models of graded burns. To assess the impact of reduced measurement dimensionality on diagnostic performance, we compared classifications derived from a research-grade SFDI system (Reflect RS) with those obtained using a simplified commercial SFDI platform (Clarifi RS). Both systems were used to image graded burns 24 hours after injury. Pixel-level classifiers were trained using regions defined by 28-day healing outcomes, and models based on the full Reflect dataset were compared with classifiers generated from reduced Reflect feature sets and datasets designed to mimic Clarifi acquisition features. Performance was evaluated using leave-one-subject-out validation. The full Reflect dataset achieved the highest classification performance, with mean F1 scores approaching 0.88. Although reducing the number of measured features decreased classification accuracy, simplified models and Clarifi-based datasets maintained F1 scores greater than 0.8 for binary classification. These findings indicate that dimensionality reduction produces a measurable but manageable loss in performance and support the development of clinically practical, application-specific SFDI systems for burn assessment.

## Full Text Availability

The license terms selected by the author(s) for this preprint version do not permit archiving in PMC. The full text is available from the preprint server.

